# Alternative hybrid access in endovascular neurosurgery: Scoping review and technical considerations

**DOI:** 10.1177/15910199241282352

**Published:** 2024-09-16

**Authors:** Matias Costa, Juan Vivanco-Suarez, Sean O’Leary, Preston D’Souza, Ravi Nunna, Sabino Luzzi, Daniel Casanova-Martinez, Akshal Patel

**Affiliations:** 1 Department of Neurosurgery, 12338 The University of Texas Medical Branch at Galveston, Galveston, TX, USA; 2 Department of Neurology, 21782 The University of Iowa Hospitals and Clinics, Iowa City, IA, USA; 3 Neurosurgery Unit, 2628 University of Missouri System, Columbia, MO, USA; 4 Department of Clinical-Surgical, Diagnostic, and Pediatric Sciences, 19001 The University of Pavia, Pavia, Lombardia, Italy; 5 Neurosurgery Unit, Department of Surgical Sciences, 18631 Fondazione IRCCS Policlinico San Matteo, Pavia, Lombardia, Italy; 6 Ecuela de Medicina, 37616 Universidad de Valparaiso School of Medicine, San Felipe, Chile; 7 Laboratorio de Neuroaatomia Microquirurgica (LaNeMic), Facultad de Medicina, Unversidad de Buenos Aires, Buenos Aires, Argentina; 8 Cerebrovascular Neurosurgery, Swedish Medical Center, 214855 Swedish Neuroscience Institute, Seattle, WA, USA

**Keywords:** Endovascular, cerebrovascular, neurosurgery, arterial access, anatomical variations, embolization

## Abstract

**Background:**

The common femoral artery and radial artery constitute the most common access sites in contemporaneous endovascular neurosurgery. Oftentimes, it may be impossible to reach the vascular target safely due to different circumstances, necessitating alternative approaches. We aim to review these “last resource” access sites described in the literature, focusing on the technical aspects as a convenient reference.

**Methods:**

We performed a comprehensive literature search of PubMed capturing articles from 1958 to 2022.

**Results:**

We identified nine alternative approaches for endovascular access, after excluding direct carotid stick: superficial temporal artery access in 60 patients, calvarial foramina access in five patients, occipital artery access in seven patients, middle meningeal artery access in six patients, vertebral artery access in 23 patients and external carotid artery, internal maxillary artery, facial artery or lingual artery in 40 patients. Indications for the use of alternative access points included tortuous or occluded vessels, small vessel calibers, or anatomic variation. Pathologies treated included dural arteriovenous fistulas, arteriovenous malformations, intracranial aneurysms, acute stroke, and intracranial stenosis. Diagnostic brain angiograms were also performed.

**Conclusions:**

Alternative vascular access routes expand the proceduralists' toolkit, enhancing the capability to manage complex cerebrovascular interventions. This review advocates for a broader understanding and consideration of these techniques, given their potential to significantly increase treatment options in neuroendovascular surgery.

## Introduction

The advent of endovascular neurosurgery has been a marked cornerstone for treating neurological pathologies. The common femoral artery (CFA) and radial artery (RA) are frequently used access vessels. In addition to anatomic familiarity, the use of large-bore catheters and the availability of percutaneous closure devices or pressure-designed bands make these approaches favored.

However, several drawbacks are reported with these access vessels. Complications with CFA are approximately ten times more common than any complication of diagnostic angiography or intervention.^1^ These include groin hematomas, arterial dissections, prolonged wound healing, and extended length of stay at the hospital.^2^ Additionally, occlusion of both CFAs represents around 2%–6% of procedural failures.^3^

Complications associated with trans-radial access (TRA) also encompass asymptomatic thrombosis of the radial artery, radial vasospasm, and the development of pseudoaneurysms. Although rare, complex regional pain syndrome^4^ and compartment syndrome^5^ have been reported.

On numerous occasions, the safe attainment of the vascular target becomes impossible due to anatomical factors or lesions at the local access site, tortuosity of the aortic arch or its branches, stenosis of the ostia, the presence of perilous collaterals along the path to the target, or neurovascular functional characteristics that hinder a secure approach.

The question arises of what to do when CFA/TRA approaches prove inadequate. Alternative access routes, such as direct puncture of the brachial and carotid arteries, are available options that have been described by Alvarez-Tostado et al.^6^ and Requejo et al.^7^ This article will elaborate on less conventional access methods, via literature search and anatomical explanations to provide practical descriptions that can be used as a reference.

## Materials and methods

### Information sources and search strategy

We performed a comprehensive review of the English language literature documenting alternative vascular access sites for cerebrovascular neurosurgery. A literature search using PubMed (US National Library of Medicine) database was performed from 1958 to 2022, with the following keywords and Boolean operators: (endovascular[tiab] OR neurovascular[tiab]) AND (punctures[mesh] OR punctures[tiab] OR “embolization, therapeutic”[mesh] OR “therapeutic embolization”[tiab]) AND (“middle meningeal artery”[tiab] OR “occipital artery”[tiab] OR “superficial temporal artery”[tiab] OR “vertebral artery”[tiab] OR “external carotid”[tiab]). Additional references were also found in the cross-check of the references of the selected articles, textbook chapters, and external sources. This study did not require the approval of the Institutional Review Board/ethics committee due to the lack of patient involvement. Since this is a scoping review, registration in the PROSPERO database of reviews was not required.

### Eligibility criteria

The inclusion criteria for this study were as follows: (1) Only studies published in the English language were considered. (2) Studies that reported on patients who had undergone an endovascular procedure (both diagnostic and therapeutic) through arteries originating from the territories of the right and left common carotid arteries, as well as the right and left vertebral arteries, were included. (3) Observational studies encompassing prospective and retrospective cohorts, case series, and case reports were eligible for inclusion. In cases where there were overlapping cohorts, we selected the cohort with the highest number of participants for inclusion in our analysis. (4) Direct carotid artery and brachial artery access were excluded.

### Data collection process

In the present study, data extraction was carried out by two independent reviewers, namely MC and JVS, using a standardized electronic form. The extracted data encompassed various aspects such as publication year, number of patients included, age and gender distribution, pathology type and location, arterial access site, rationale for alternative endovascular access, technical details of the procedure, procedural success, treatment type (in cases where access was obtained for therapeutic purposes), procedural outcomes, and complications.

## Results

34 studies, encompassing 134 patients, were reviewed. The mean age was 51.3 years, with ages ranging from 16 to 86, based on 26 studies that reported this data. Among the 25 studies that included sex information, there were 28 females and 18 males.

**Figure 1.**
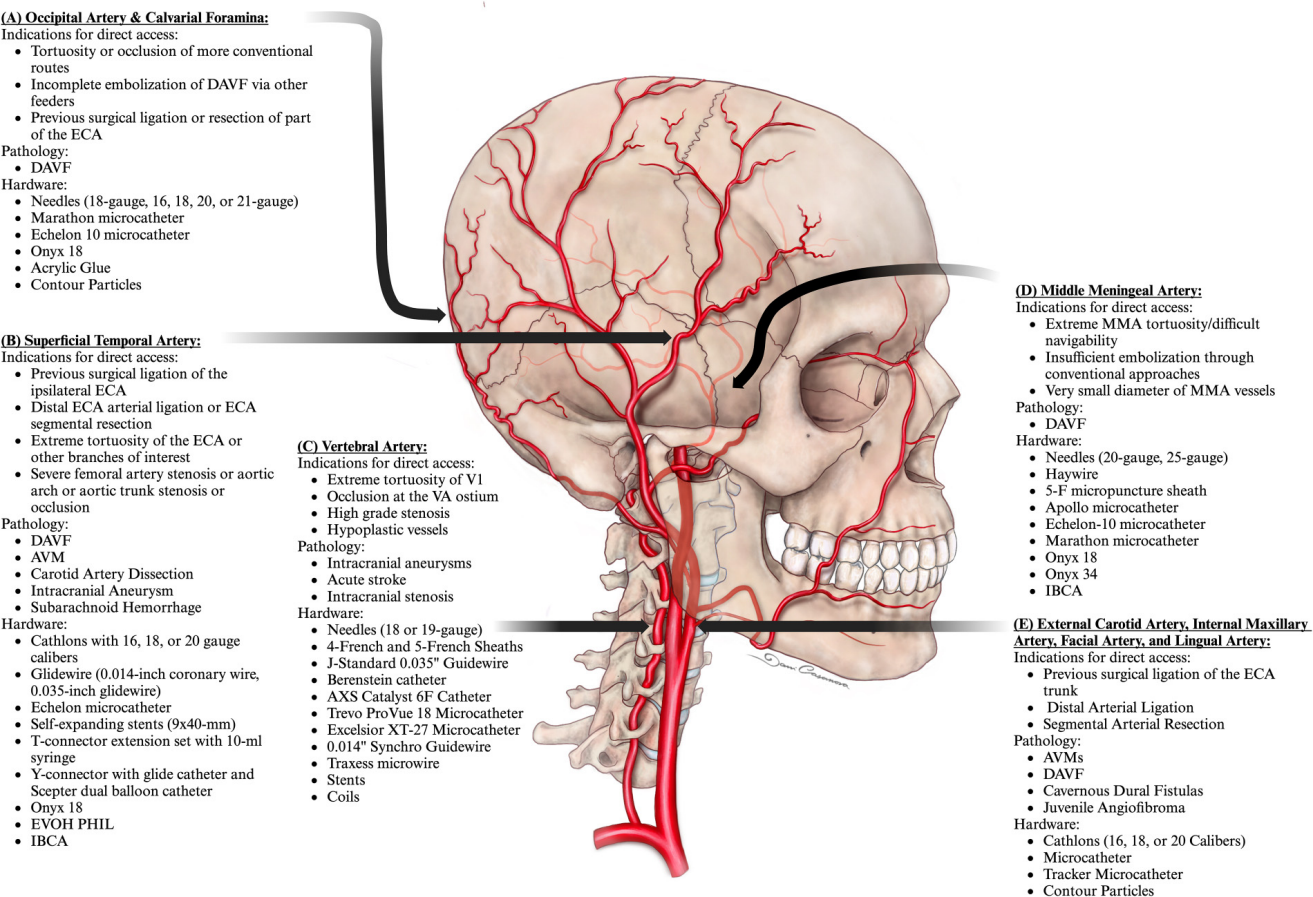
Indications, pathology, and utilized hardware for alternative access points including occipital artery and calvarial foramina (A), superficial temporal artery (B), vertebral artery (C), and middle meningeal artery (D), and (E) external carotid artery with accessed branches including the internal maxillary artery, facial artery and lingual artery.

Alternative direct access sites included the superficial temporal artery (STA),^8–18^ occipital artery (OA),^13,19–21^ middle meningeal arteries (MMAs),^14,18,22–25^ vertebral artery (VA),^4,26–40^ calvarial formina,^19,20^ and the external carotid artery, internal maxillary artery, facial artery and lingual artery^13^ (Figure 1). Approaches included percutaneous (direct needle stick),^4,8–18,21,23,26–41^ transcranial (craniotomy followed by needle stick),^19,22,24^ and transforaminal techniques (needle stick through natural skull foramina).^19,20^

Indications for alternative access included target vessel tortuosity (35.3%),^4,8,10,11,19,20,22,23,31–33,41^ incomplete embolization through a conventional access (17.6%),^17,24,34,36,38,39^ safer access planned through alternative approach than conventional access allowed (8.82%),^21,26,35^ unsuccessful vessel puncture (8.82%),^27,29,30^ extremely small vessel calibers (8.82%),^18,28,33^ target vessel ostium stenosis (5.88%),^39,40^ embolic material not reaching target due to extreme vessel tortuosity or previous surgical ligation of target vessels (5.88%),^9,13^ occlusion obstructing transfemoral approach (2.94%),^18^ dangerous anastomoses on the way (2.94%),^14^ impossibility of target vessel catheterization with incomplete circle of Willis for transcirculation approaches (2.94%),^37^ and access for diagnostic brain angiography (8.82%).^12,15,16^

Pathologies treated included intracranial aneurysms (IAs) (14.18%, 19 patients),^4,12,15,16,27,31,33–36,38,39^ arteriovenous malformations (AVMs) (28.36%, 38 patients),^13,15^ dural arteriovenous fistulas (DAVFs) (11.19%, 15 patients),^8,9,13,14,19–24,41^ intracranial stenosis or occlusion (3.73%, five patients),^10,11,26,37,40^ stroke (2.88%, four patients),^28–30,32^ cavernous dural fistula (2.99%, two patients),^13^ and juvenile angiofibroma (2.34%, three patients).^13^

### Superficial temporal artery direct access

Eleven studies that included a total of 60 patients were identified in which the STA was used for direct vascular access.^8–18^

#### Relevant anatomy

The STA originates from the internal maxillary artery, situated inferior to the zygomatic process. Its point of origin is located above the parotid gland region, near the temporal and zygomatic branches of the seventh cranial nerve. These two nerve branches run anterior to the artery. At this juncture, the STA runs in a cranial and superficial direction, and its pulsations can be easily palpated. It divides ∼ 2–5 cm above the zygoma into a frontal branch and a parietal branch, with variable trajectories that either run anteriorly or towards the parietal region, respectively. The distance between the zygomatic process and the bifurcation of the common carotid artery is ∼ 10 cm. If necessary, a cannula can be advanced this distance, as will be further described.

#### Indications

STA utilization covered several pathologies, including DAVF embolization,^8,9,13,14^ skin or subcutaneous AVM embolization,^13^ retrograde common carotid artery (CCA) stent placement^10^ retrograde internal carotid (ICA) stent placement^11,42^ and retrograde brain angiographies.^12^ Factors contributing to the selection of the vessel included previous surgical ligation of the ipsilateral external carotid artery (ECA) trunk, distal ECA arterial ligation or ECA segmental resection,^13^ extreme tortuosity of the ECA or other branch of interest^9,14^ and severe femoral artery stenosis or aortic arch or aortic trunks stenosis or occlusion.^42^

#### Technique

Due to the possibility of spasms occurring in the artery during manipulation or puncture, careful planning is advised to minimize the number of attempts made to gain STA access. Vessel localization can be achieved under fluoroscopy via the road map technique for needle guidance^9,13^ or by stereotactic computed tomography angiography (CTA) volumetric scan incorporated into the frameless navigation system.^14^

Arterial access can be obtained through percutaneous^8,9,13^ or open surgical exposure of the STA^10–12,14,16,17,38^ and puncture via cathlons (Critikon, Chatenay, France) with 16-, 18-, or 20-gauge calibers, determined by the estimated vessel size.^9,10,12,13,38^

Embolization procedures are performed by direct injections through the cathlon, as well as using the cathlon as a sheath for a microcatheter.^13^ Further techniques included feeding the microcatheter Echelon (Medtronic, Minneapolis, Minnesota, USA) directly into the STA,^14^ or with a glide catheter connected to a Y-connector and a Scepter dual balloon catheter through it and inflated before embolization.^9^ Embolization materials included Onyx 18 through an Echelon microcatheter,^14^ ethylene vinyl alcohol copolymer (EVOH, PHIL 25%) through a Scepter dual balloon catheter (Microvention, Tustin CA)^9^ or isobutyl 2-cyanoacrylate (IBCA) in the case of Barnwell et al.,^8^ in which the microcatheter used was not described.

Stenting techniques through STA access have been described for carotid dissection.^10^ An 18-gauge needle was used for access after STA exposure through a 2-cm skin incision, a 0.014-inch glide wire, and a 5-f diagnostic catheter navigated downstream, angiography was performed, and a 9 × 40-mm self-expanding stent was deployed. Matsubara et al.^38^ used a similar access technique to the STA, but a 0.035-inch glidewire was used to get to the CCA. Following this, a special snare technique through the right brachial artery was used to bend the system back towards the ICA where a stent was deployed. Ivancev et al*.*^11^ used a similar access to the previous two authors, but a 0.014-inch coronary wire was used and a microcatheter was loaded over the wire.

Trans-STA retrograde angiography has also been described in the literature during open surgery for IA clipping. After 2 cm of STA is surgically exposed, a 20 or 18-gauge intravenous catheter can be used for cannulation.^12,15,16^ This can be connected to a T-connector extension set with a 4-inch microbore sidearm and a 10-mL syringe.^12,15,16^ The entire system is prefilled with 5000 units of heparin in 1000 mL solution and the catheter secured to the adjacent galea aponeurotica or deep temporal fascia with a 3-0 suture.^12^ Similarly, retrograde CCA and ICA procedures have also been performed after STA access. According to Cummins et al*.*,^15^ resistance will be felt when a cannula is advanced caudally for 5 or 6 cm and backflow will be lost. At this point, they recommend withdrawing the cannula for 1–3 cm and advancing it again caudally over a wire. If the STA is accessed for angiography via the CCA, 10 cc of contrast often suffices. Further recommendations of trans-STA approaches involve approaching the STA as proximal as possible to avoid the artery's sharp turn when traversing the zygomatic process.^17^

Adams et al.^16^ performed trans-STA angiographies in 28 patients with IA, 26 of which presented with subarachnoid hemorrhage (SAH). They exposed the STA during the primary surgery, inserted a catheter and guidewire, and secured them < 10 cm from the zygomatic arch. The sheath was kept in place under continuous heparinized irrigation, allowing intraoperative and serial postoperative angiographies to be performed. The catheter was additionally flushed to push any debris into the ECA system preceding angiographic runs.^16^ Cummins et al.^15^ also used a similar technique during 25 cerebrovascular open cases, for intra and post-operative brain angiographies.

#### Outcome

Complications included one subcutaneous hematoma that resolved spontaneously,^13^ one frontal lobe infarct in a case of a borden IIa + b fistula (not related to the access, but an embolic phenomenon),^14^ and one case of skin necrosis with infection in the area of EVOH injection that resolved completely.^9^ In all cases, the therapeutic goal was achieved.

### Calvarial foramina

Two studies were identified, involving a total of five patients, in which these foramina were utilized for direct vascular access.^19,20^

#### Relevant anatomy

**Figure 2.**
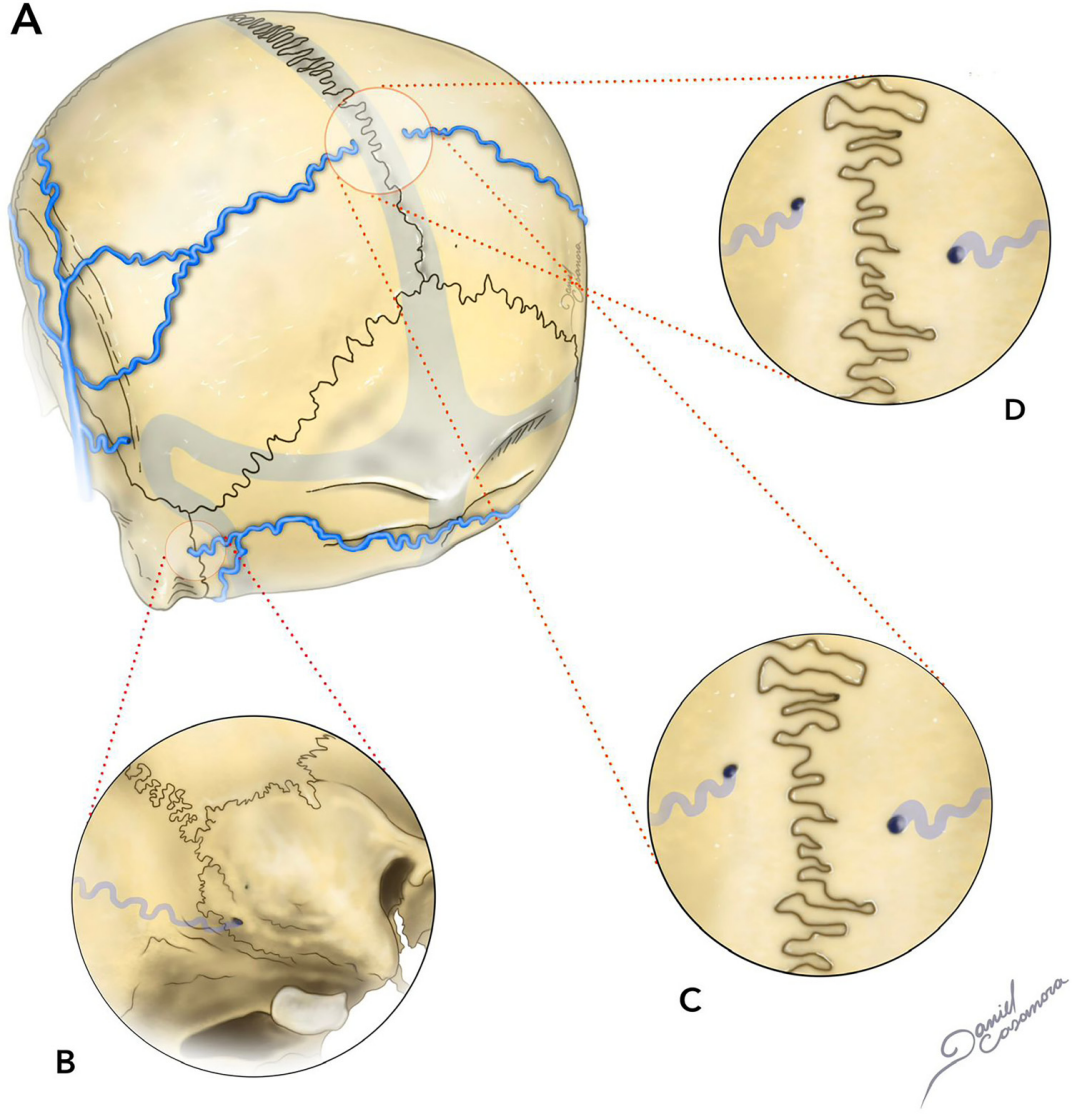
Illustration of adult skull (A) with mastoid foramina (B) as well as small parietal foramina, anterior to the lamboid suture and lateral to the sagittal suture (D, C).

Parietal foramina are two round, diminutive, defects in the cranial vault, located close to the midline on the parietal bone. They are 1–2 mm in diameter and are present in 60%–70% of normal adults (Figure 2).^43^ They represent normal anatomical variations, and usually transmit *emissary veins* draining into the superior sagittal sinus. They can be found unilaterally or bilaterally on the posterior region of the *parietal bone*, about 1–2 cm from the midline.^19,20^

A branch of the *OA* may sporadically be accessed through these foramina. In very rare circumstances, large or even giant parietal foramina can be found. Mastoid foramina is present in around 90% of the cases.^44^ They are variable in size, number, and position, being commonly located near the posterior margin of the mastoid process, within the temporo-occipital suture. Different vascular structures can be accessed through this foramen, including the mastoid emissary vein (connecting posterior auricular vein-sigmoid sinus) and a branch of the occipital artery, usually the posterior meningeal artery.^19,20^

#### Indications

The reason for alternative access use was marked elongation of the arteries ascending to the fistula and the tortuosity of the vein by Gonzalo et al.^19^ and tortuosity of the OA by Chapot et al.^20^ The pathology treated in all cases was the treatment of DAVFs.

#### Technique

The localization of the puncture site was conducted using angiographic runs, fluoroscopy, and roadmaps in all cases. A direct puncture was carried out using an 18-gauge needle in all cases, and the microcatheters utilized were Marathon (Medtronic, Minneapolis, Minnesota, USA) and Echelon-10 (Medtronic, Minneapolis, Minnesota, USA). Generally, the procedures were performed with the patient in either the prone or supine position, depending on the position of the foramina and the patient's characteristics. AP and lateral fluoroscopy views were employed to identify the location of the foramina and to monitor the needle's progress.^19,20^

Chapot et al.^20^ emphasize the importance of ensuring that the needle tip does not breach the inner table of the skull to minimize the risk of intracranial bleeding.

#### Outcome

In all cases, access was successful, the therapeutic target was achieved and there were no complications related to the procedure.

### Occipital artery direct access

Four studies with seven patients were identified, describing direct access to the occipital arteries.^13,19–21^

#### Relevant anatomy

The OA commonly originates from the posterior aspect of the ECA in the neck. It follows a posterior course and passes through a groove on the temporal bone, located medial to the digastric muscle and the mastoid process. It continues its path towards the external occipital protuberance and eventually reaches the scalp. At the level of the cranial insertions of the trapezius and sternocleidomastoid muscles, it travels through the trapezius muscle and the nuchal fascia. During its course, the OA gives rise to several trunk branches before generating terminal branches. Notably, these trunk branches include muscular branches, the stylomastoid artery, and meningeal arteries that supply the dura mater of the mastoid process and the diploë. Additionally, it enters the mastoid foramen alongside an emissary vein. Of particular significance, the OA establishes crucial anastomoses along its trajectory, including those with the VA. These are particularly important during embolization procedures since uncontrolled forward injections can evolve into brainstem strokes through retrograde embolization of the VA.

#### Indications

Only DAVFs were treated this way. Indications to choose this access included tortuosity or occlusion of more conventional arterial or venous routes,^19^ incomplete embolization of DAVF via other feeders,^20^ and previous surgical ligation or resection of part of the ECA.^13^ Gensburg and Radford^21^ considered the direct OA approach the safest and least invasive due to the patient’s advanced age and aim towards symptomatic relief only, and not a complete cure for DAVF.

#### Technique

Gonzalo et al.^19^ mentioned a prone position for access. We believe the prone position to be inconvenient if a control digital subtraction angiography (DSA) through the femoral or radial routes is necessary. In these circumstances, insertion of a conventional radial or femoral long sheath before turning the patient prone can be an option.

One method to localize the OA is an angiographic roadmap under fluoroscopy for needle guidance.^19,20^ Additionally, direct palpation and percutaneous access have also been described, starting 4 cm behind the ear where the artery is most superficial and palpable.^13,21^ Only one author performed bilateral OA access.^21^ Parietal and mastoid foramina, as previously discussed, offer potential conduits for anastomosis involving the occipital and posterior meningeal arteries. These anastomoses may be hypertrophied in DAVF, and direct puncture through these foramina may allow access to arteries feeding DAVFs^20^ OA feeders to DAVFs were accessed through the parietal foramen (PF) in one case^19^ and the mastoid foramen in another one.^20^ Vessel puncture was carried out via an 18-gauge needle in two studies.^19,20^ Gobin et al.^13^ described the use of 16, 18, or 20-gauge needles according to the situation. Gensburg and Radford^21^ used 21-gauge needles. Embolization procedures were done through Marathon microcatheters^19^ and Echelon 10 microcatheters catheters.^20^ One author described direct embolization through the cathlon or using the cathlon as a sheath for a microcatheter.^13^ Embolic materials used included Onyx 18,^19^ acrylic glue (Glubran 2, GEM Srl)^20^ and contour particles (Interventional Therapeutics Corp., South San Francisco, CA).^21^

It is important to remark that, with transforaminal approaches, one must consider the risk of an epidural hematoma should the needle advance beyond the inner table of the calvaria.^20^ Attention to OA and VA anastomosis must be considered for embolization or else forward injection of material can result in brainstem strokes.

#### Outcome

There were no major complications associated with these approaches. Gobin et al.^13^ reported a self-resolving local hematoma. The therapeutic target was achieved in all cases.

### Middle meningeal artery direct access

Direct MMA access was described in six studies with six patients with DAVF pathologies.^14,18,22–25^

#### Relevant anatomy

The MMA is a significant branch of the ECA and typically originates from the internal maxillary artery (IMAX). It courses cranially from its origin and traverses the foramen spinosum at the base of the skull, giving rise to cavernous and petrosal branches. Distally, it divides into a frontal or anterior branch, a parietal or posterior branch, and a petrosquamosal branch. These branches primarily supply the convexity duramater but also contribute to the vascularization of the falx cerebri.

#### Indications

Reasons for direct MMA access included extreme MMA tortuosity, difficult navigability,^22–25^ insufficient embolization through conventional approaches,^24^ or very small diameter of the MMA vessels, making other approaches impossible.^18^

#### Technique

When performing embolization through the MMA, it is crucial to conduct an early study and identification of its potentially hazardous anastomoses. These include the anterior branch with the ophthalmic artery or its branches, the petrosal branch with the facial nerve arcade, and the petrosquamosal branch with branches of the anterior inferior cerebellar artery (AICA) or ascending pharyngeal artery (AphA).

Target vessels were localized and accessed by using fluoroscopy and angiographic roadmap guidance,^24,25^ direct craniotomy over the estimated pterional area with naked eye localization of the MMA,^14,18^ craniotomy and drill decortication over MMA,^14,18,23–25^ and CT-angiography guided stereotactic navigation.^23^ In one case, the posterior meningeal artery was accessed through the mastoid foramen.^20^ Vessel access was achieved with a 20-gauge needle, hairwire, 5-F micropuncture sheath, and Echelon-10 microcatheter,^24^ a sharp nick over the MMA parietal branch and an Apollo microcatheter insertion over a microwire, followed by sutures around the catheter for anchorage,^25^ a stand-alone Marathon microcatheter,^14^ or a 25-gauge needle, microwire and an Echelon-10 microcatheter.^23^ In our experience, the sheath or microcatheter used to cannulate the MMA can be anchored to the duramater itself with a suture around the entry point, and another one on a more proximal portion of the hardware to the skin in order to avoid kinking of the system. Verapamil has been described for injection through a control sheath (i.e. the catheter used to perform the angiographic roadmap guidance) to help dilate the target artery in case of spasm or if smaller than expected arterial size as described in one case.^24^ Catheterized portions of the MMA reported include the main MMA trunk,^14,23,24^ both anterior and posterior branches^18^ or the posterior branch alone.^25^ Embolization material used included Onyx 18,^14,24,25^ Onyx 34^23^ N-butyl 2-cyanoacrylate (NBCA),^22^ and IBCA.^18^ Again, care must be taken to identify potentially hazardous anastaomses between MMA and ophthalmic arteries, facial nerve arcades, and AICA/AphA.

#### Outcome

In all cases, the treatment goal was achieved and there were no complications related to the approach.

### Vertebral artery direct access

A total of 16 papers were identified, which described 23 patients who underwent direct access to the VA.^4,26–40^

#### Relevant anatomy

The VA is anatomically divided into four distinct segments, commonly referred to as V1–V4. The initial segment, V1, originates from the subclavian artery and travels in a dorsal direction until it reaches the foramen of C6. At this point, the V2 segment begins and extends from the transverse foramina of C6 to C2. The subsequent segment, V3, is characterized by its condensed and convoluted nature. In this segment, the artery enters the transverse foramen of the C1 before changing direction and bending medially to pass behind the lateral bulk of the corresponding vertebrae. From this point, the artery takes a sharp turn to penetrate the dura mater, giving rise to the V4 segment, and enters the cranium through the foramen magnum.

#### Indications

The pathologies addressed in these studies were diverse, including intracranial aneurysms, acute stroke, and intracranial stenosis, with IA treatment being the most common indication, as reported in 16 patients.^4,27,31,33–36,38,39^ In all cases, traditional approaches were attempted first, but failed due to various reasons. The most frequent cause of failed TRA or trans-femoral access (TFA) catheterization was extreme tortuosity in the V1 segment of the VA.^4,26–29,31–34,38,39^ Occlusion at the VA ostium, high-grade stenosis, or hypoplastic vessels were other cited reasons for difficult catheterization.^30,31,33–35,37,38,40^

#### Technique

Both open and percutaneous techniques were described for direct access to this artery. Open techniques generally consisted of neck dissection with exposure of the V1 segment of the VA^27,34,38^ within the supraclavicular fossa. The patient was positioned supine with the neck in extension. The skin was incised along the anterior border of the sternocleidomastoid muscle with sharp and blunt dissection carried deep towards the carotid sheath, which was mobilized laterally. The V1 segment was then identified medial and deep to the carotid sheath and further exposed as it entered the C6 transverse foramen. Sekhon et al.^40^ also reported a suboccipital/far-lateral approach for exposure of the V3 segment and catheterization prior to its intradural transition. Reported percutaneous approaches included both ultrasound and fluoroscopy-guided techniques to access the V2 and V3 segments. Semeraro et al.^28^ reported an ultrasound-guided technique consisting of supine positioning with neck extension and a posterolateral approach for direct needle puncture in the V2 segment between the transverse foramen of C4 and C5, while O’Reilly et al.^30^ positioned the ultrasound probe just inferior to the mastoid process for direct puncture of the V3 segment. The most frequently used percutaneous approach was a combination of roadmap technique guidance with TFA or TRA angiography and direct needle puncture.^26,28–31,34^ Weill et al.^39^ also reported the direct palpation of the fascial plane between the brachiocephalic vessels and the trachea. A needle was then introduced medially until contact was made with the C4 vertebral body. The needle was then laterally re-directed and advanced in between the transverse foramina into the VA for access. We consider this philosophy to be highly obsolete and irrelevant nowadays where navigation and ultrasound technologies are so highly developed.

Irrespective of the chosen approach, the use of an 18- or 19-gauge needle was commonly observed. Among the various options available, most studies indicated the utilization of either four French or five French sheath systems. Following the introduction of a sufficient sheath into the artery, the majority of studies reported the direct use of a microcatheter and microwire. Once arterial access is safely achieved through an alternate access approach, multiple options for subsequent equipment decisions during the procedure, such as embolization (using Onyx, particles, or n-BCA), stents, or coils have been described.^27,34,38^

#### Outcome

Complications associated with direct VA access reported included contrast extravasation with subsequent improvement.^33^ vasospasm, and transient neurologic deficit.^36^ It is worth noting that no cases of hematoma or VA occlusions were reported as complications of direct VA access.

### External carotid artery, internal maxillary artery, facial artery, and lingual artery

One retrospective paper was identified which included percutaneous puncture of the external carotid artery, internal maxillary artery, facial artery, and lingual artery, which described 40 patients.^13^

#### Relevant anatomy

The ECA branches from the ICA, then further split into six branches, including the internal maxillary, facial, and lingual arteries. These branches provide collateral to the ICA and vertebral arteries. The IMAX originates posteriorly to the mandibular neck, traverses over the parotid gland, and courses between the pterygomandibular ligament and mandibular ramus, supplying blood to the deep facial structures including the maxilla and mandible. The facial artery runs beneath the posterior aspect of the submandibular gland then curves upwards over the mandible's body, and follows along the anteroinferior margin of the masseter muscle, providing blood to the superficial structures of the face. The lingual artery courses medially adjacent to the greater horn of the hyoid bone, then descends deep between the gastric and stylohyoid muscles, traversing between the middle constrictor of the pharynx and the hyoglossus muscle anteriorly to the hyoglossus muscle's border, providing blood supply to the tongue and the floor of the mouth.

#### Indications

The pathologies treated by Gobin et al.^13^ in these arteries included 33 facial AVMs, four direct ECA AVMs, one DAVF, two cavernous dural fistulas, and three juvenile angiofibromas. Notably, three of these 43 pathologies involved STA access, though this specific data was not separately detailed by pathology. Indications for alternative access included previous surgical ligation of the ECA trunk, a more distal arterial ligation, or a segmental arterial resection.

#### Technique

For all percutaneous punctures, first, an angiogram was used to visualize arterial connections, with a puncture then being performed under fluoroscopy guidance. The ECA was punctured laterally at its origin avoiding puncture of the internal carotid artery. Embolization was either through the cathelon, which was used with 16, 18, or 20 calibers, as a sheath, through a microcatheter (Pursil, Bait-Co, Montmorency, France), or through a Tracker microcatheter (Target Therapeutics, San Jose, Calif) with the use of contour particles.

The puncture for the IMAX was made at its origin, behind the neck of the mandible, with an entry point under the lobe of the ear, angling the needle medially, anteriorly, and upward at 45°. The facial artery was accessible through three different approaches: its origin, its submandibular course, and distally on its superficial jugal course. Finally, the lingual artery was punctured close to its origin, on its first curve with a superior convexity, above the superior edge of the great horn of the hyoid bone.^13^

#### Outcome

The ECA was successfully accessed on the first attempt in 31 cases. For the internal maxillary artery, punctures were successful on the first attempt in six cases, with two additional successes on a second attempt, and one remaining unsuccessful. In the case of the facial artery, seven initial punctures were successful, two more achieved success in a subsequent session, and one was unsuccessful. Lastly, for the lingual artery, six initial punctures succeeded, two were successful during a second session, and one failed to achieve success.^13^

Complications included site hematomas in eight cases that resolved spontaneously, and inadvertent puncture of the ICA in six cases that were planned for the ECA.^13^

## Discussion

The majority of neurointerventional procedures globally are performed either by the TFA or, more recently, the TRA routes. The Standards and Guidelines Committee of the Society of NeuroInterventional Surgery (SNIS) notes that TFA is the traditional approach and performed in over 95% of cases while maintaining support for TRA as an alternative access site due to a number of retrospective series with promising results.^45^ However, even the consensus report notes that alternative sites such as direct carotid or VA access may be necessary when the anatomy precludes a more traditional access site, noting the possible increased risk of procedural complications with alternative access sites. While traditional access sites are frequently preferred, multiple reasons may arise that necessitate the use of a last-resort vascular access site.

Besides direct carotid sticks and brachial artery access, which were not addressed due to the vast literature already available on the topic, our scoping review has identified alternative access sites as the VA, STA, OA, MMA, ECA and its branches and access via calvarial foramina. Each of these has a number of anatomic and technical considerations critical to successful implementation which have been comprehensively detailed in the current study. Importantly, the materials selected, and the caliber of catheters must be tailored to the regional anatomy and target vessel characteristics.

A number of advantages can be recognized in the use of direct access techniques. Direct access obviates the need for catheter manipulation in the aortic arch and vessels of the neck as well as bypassing anatomic barriers such as peripheral vessel occlusion or profoundly tortuous vasculature. In theory, a reduction in time navigating through the difficult aortic arches and vessels of the neck may be associated with a lower risk of ischemic stroke secondary to thromboembolic complications from vessel navigation. Notably, this perceived benefit has not been comprehensively investigated sufficiently in prospective studies. Another theoretical advantage may be found in the physics of transmission of torque and forces. Direct alternative access sites are generally in much closer proximity to the pathology, allowing for excellent maneuverability of microwires and microcatheters. Additionally, in cases where the target vessel is clamped or sacrificed proximal to the puncture site, the technique also allows for embolic material reflux control.

The implementation of these techniques must be carefully considered in light of potential procedural complications that may arise. One of the most common technical challenges is the anchoring and stabilization of sheaths. Empirical evidence from the senior author's institution, as well as case series, suggests that a combination technique involving tunneling of the catheter and suturing it directly to the skin offers the most robustness and reliability. Another drawback is the limited availability of suitable materials. Many wires and catheter platforms are designed for use via TRA and TFA routes, and are not well-suited for use in close proximity to the pathology. This increases the likelihood of potential hardware disruption or complete dislodgement.

Direct access techniques are invasive and may require the presence of a neurosurgeon, which may not always be feasible in regular healthcare systems. It may also result in increased radiation exposure, particularly in the hands of the operator. Additionally, catheter compatibility with dimethyl sulfoxide (DMSO) must also be taken into account. Most materials used for direct access are not compatible with DMSO. A safer approach would involve the insertion of a DMSO-compatible microcatheter within a non-DMSO-compatible sheath. This would partially protect the sheath wall from contact with DMSO, thereby mitigating the risk.

Finally, the infrequency of direct access use and the technical challenges associated with it should be considered for the clinician. Surgeons and interventionalists may need to overcome a theoretical increase in procedure risk due to the low volume and repetition of these techniques, as well as an interventional team of surgical technicians, anesthesiologists, and nurses who may not be familiar with their successful implementation.

## Conclusion

Alternative access strategies must be considered when conventional approaches are not feasible or insufficient to treat the pathology. Such examples include access through the STA, MMA, occipital artery, vertebral artery, calvarial foramina, and ECA tributaries, each with their own technical considerations. Knowledge of these approaches widens the armamentarium of the proceduralist and lends to the ever-changing philosophy of cerebrovascular interventions.
